# Equivalent outcomes in nasal symptoms following microscopic or endoscopic transsphenoidal surgery: results from multi-centre, prospective study

**DOI:** 10.1007/s00701-022-05138-5

**Published:** 2022-02-08

**Authors:** Charlie Osborne, Daniel Lewis, Ben Dixon, Carmela Caputo, Alison Magee, Kanna Gnanalingham, Yi Yuen Wang

**Affiliations:** 1 Department of Neurosurgery, St Vincent’s Hospital, Melbourne, VIC Australia; 2 Department of Neurosurgery, Manchester Centre for Clinical Neurosciences, Salford Royal NHS Foundation Trust, Manchester Academic Health Science Centre, Manchester, UK; 3 Department of Ear, Nose & Throat, Head and Neck Surgery, St Vincent’s Hospital, Melbourne, VIC Australia; 4 Department of Endocrinology, St Vincent’s Hospital, Melbourne, VIC Australia; 5 Keyhole Neurosurgery, Melbourne, VIC Australia

**Keywords:** Nasal symptoms, Transsphenoidal surgery, Endoscopic, Microscopic

## Abstract

**Background:**

T ranssphenoidal surgery (TSS) is the standard approach for resection of pituitary lesions. Historically, this has utilized the microscopic approach (mTSS); however, the past decade has seen widespread uptake of the endoscopic approach (eTSS). The purported benefits of this include improved visualization and illumination, resulting in improved surgical and endocrinological patient outcomes. It is also believed that eTSS results in fewer post-operative nasal symptoms compared to mTSS; however, few papers have directly compared these groups.

**Objectives:**

We sought to compare nasal symptoms after endoscopic uninostril (eTSS-uni), endoscopic binostril (eTSS-bi) and microscopic endoscopic transsphenoidal surgery (mTSS).

**Methods:**

The General Nasal Patient Inventory (GNPI) was prospectively administered to 136 patients (71 non-functioning adenomas, 26 functioning adenomas, 39 other pathology) undergoing transsphenoidal surgery at multiple time points (pre-operatively; days 1, 3 and 7–14; months 1, 3 and 6 and 1 year post-operatively). All surgeries were performed by subspecialist pituitary surgeons in three subgroups — mTSS (25), eTSS-uni (74) and eTSS-bi (37). The total GNPI scores (0–135) and subscores for the 45 individual components were compared across three groups assessing for temporal and absolute changes.

**Results:**

Irrespective of surgical approach used, GNPI scores were significantly higher on post-operative day 1 (*p* < 0.001) and day 3 (*p* ≤ 0.03) compared to pre-treatment baseline (mixed-effects model). By 1 month post-operatively, however, post-operative GNPI scores were no different from pre-treatment (*p* > 0.05, mixed-effects model). Whilst the eTSS-uni group demonstrated significantly lower GNPI scores at day 1 post-op compared to the mTSS group (*p* = 0.05) and eTSS-bi group (*p* < 0.001), there was no significant difference in post-operative scores between approaches beyond 1–2 weeks post-operatively. Similar results were obtained when the non-functioning tumour group was analysed separately.

**Conclusions:**

Transsphenoidal pituitary surgery is well tolerated. Post-operative nasal symptoms transiently worsen but ultimately improve compared to pre-operative baseline. Operative approach (microscopic, endoscopic uninostril or endoscopic binostril) only has a transient effect on severity of post-operative nasal symptoms.

**Supplementary Information:**

The online version contains supplementary material available at 10.1007/s00701-022-05138-5.

## Introduction

Endoscopic transsphenoidal surgery (eTSS) is increasingly becoming the standard approach for resection of pituitary lesions [6, 20]. Compared to the traditional microscopic transsphenoidal approach (mTSS), the endoscope is reported to afford greater visibility allowing for more extensive dissection and higher rates of gross tumour resection [1, 22, 28]. The endoscopic approach typically affords shorter operative time and reduced length of stay with comparable, if not improved, rates of post-operative complications across the two modalities [5]. Specifically, a lower rate of nasal symptoms including anosmia, septal perforations and epistaxis is described [7, 9, 17, 18, 24].

Regardless of the surgical approach, patients who undergo TSS often develop post-operative nasal symptoms such as pain, congestion, discharge, bleeding and altered or unpleasant tastes or smells. Previously, we have used the General Nasal Patient Inventory (GNPI), a 45-item patient-derived validated questionnaire, to assess the temporal changes in nasal symptoms following eTSS for pituitary lesions [3, 27]. Following eTSS, nasal symptoms typically develop early in the post-operative course and tend to resolve by a few weeks post-operatively [27]. Overall, the surgery is well tolerated and, despite a transient exacerbation, the post-operative nasal symptoms also improve when compared to pre-operative baseline [2].

In considering nasal complications following TSS, whilst several centres report on the incidence of defined anatomical complications (e.g., epistaxis and septal perforations), few papers have assessed the functional effects of nasal complications through the administration of patient-reported sinonasal QOL questionnaires [7, 16, 19, 21]. Such patient-based questionnaires are increasingly being validated for use as outcome measures, providing individualized evaluation of the success of a given therapy.

The GNPI is a sensitive tool used in assessing pre- and post-operative nasal symptoms following nasal intervention [3]. In this prospective study, we used the GNPI with the aim of comparing nasal symptoms across surgical groups of patients undergoing either eTSS or mTSS to clarify if any differences exist.

## Methods

One hundred and thirty-six consecutive adult patients undergoing transsphenoidal pituitary surgery over an 18-month period (January 2015 to August 2016) were prospectively enrolled into the study. Surgery was performed by subspecialist pituitary neurosurgeons (two endoscopic and one microscopic) across three centres. All patients had been listed for surgery after an institutional multi-disciplinary meeting. Patient demographics including clinical presentation, MR imaging findings, endocrine profile, operative details, complications and tumour pathology were noted.

Patients either underwent a microscopic approach, endoscopic uninostril approach (eTSS-uni) or endoscopic binostril approach (eTSS-bi), depending on surgeon’s preference and type of pathology (e.g., binostril approaches for larger tumours and for non-adenomatous lesions requiring extended transsphenoidal approaches).

### Operative technique

Both operative techniques have been well described previously and are summarized here [8, 13, 29]. All patients received broad spectrum peri-operative antibiotics with anaerobic cover.

### Microscopic

Nasal preparation was performed with injection of vasoconstrictor (lignocaine/adrenaline 1:100,000) into the right nasal septum and antiseptic betadine wash performed with pre-operative packing with betadine-soaked nasal ribbons. A single nostril approach is utilized with gradual expansion of the nasal cavity with nasal speculums resulting in lateralization of the middle turbinate and a posterior fracture of the bony nasal septum. In this manner, posterior nasal mucosa is opened directly and the sphenoid ostia identified. A wide unified sphenoidotomy is performed to expose the sella floor. The sphenoid mucosa is reflected laterally to allow access to the pituitary proper and tumour resection performed.

Closure following tumour removal involves inspection of the cavity to identify significant mucosal bleeding points, which are controlled with diathermy. The nasal speculum is removed and the deflected middle turbinate and nasal septum are medialized. Nasal packing is not routinely employed post-operatively and no nasal decongestant is prescribed.

### Endoscopic

Nasal preparation is performed with topical applications of pre-operative decongestants (cophenylcaine, Aurum Ltd.) and injection of vasoconstrictor (lignocaine/adrenaline 1:100,000) into the nasal septum in both nostrils. No antiseptic wash was utilized.

For the eTSS-uni approach, a left or right nostril approach was used, depending on the favourable anatomy and the site of pathology (e.g., left nostril approach for a right-sided sellar lesion). After lateral deflection of the middle turbinate and nasal septum, the posterior nasal mucosa overlying the anterior sphenoid wall was cauterized and retracted before opening the sphenoid sinus bilaterally.

For eTSS-bi approach, a right superior septal incision is made extending from the sphenoid ostium superomedially to allow for preservation of the nasoseptal flap. In cases where a high flow CSF leak is expected, harvest of the nasoseptal flap is undertaken [11]. A limited posterior septectomy with unified sphenoidotomy is then performed to achieve an adequate corridor for exposure of the sella allowing passage of surgical instruments through both nostrils.

Closure following tumour removal involved inspection of nasal cavities to identify significant mucosal bleeding points that are controlled with diathermy. Nasal packing is not routinely employed apart from bioresorbable dressing (Nasopore, Stryker) to separate the mucosal surfaces in the nasal cavity. Patients were instructed to use a topical nasal saline irrigation post-operatively, until the nasal symptoms settled.

In the event of CSF leakage, a graded operative repair was undertaken using combinations of haemostatic bioresorbable gelatine sponge and dural sealant (Duraseal, Confluent Surgical, USA) for grade 1 leaks (i.e., minor leak with no obvious arachnoid defect) [29]. In the event of a grade 2 (i.e., moderate CSF leak with visible arachnoid defect) or grade 3 CSF leak (i.e., large CSF leak with large dural defect), in addition to the above, a fat graft, dural substitute (Durafoam, Codman, UK) and/or vascularized nasoseptal flap was used on a selective basis [29].

### Nasal symptoms

The patients were asked to complete the GNPI questionnaire assessing nasal symptoms across eight time points: pre-operatively, days 1, 3 and 7–14 post-operatively and months 1, 3, 6 and 12 months post-operatively. For each of the 45 items, the patients had to select from 4 numerical answers (0 = not present, 1 = mild, 2 = moderate, 3 = severe). Total scores (0–135) for each patient and individual scores for each item were recorded and analysed allowing assessment of global as well as specific changes in nasal symptoms over time. An overview of each item included within the GNPI questionnaire is provided in supplementary table S1. Approval for the study was obtained from the local institutional review board and all patients consented to participate in the study.

### Statistical analysis

Stata version 11 and the SPSS statistical software package (version 25, IBM Corp.) were used for all statistical analyses. Descriptive results are presented as medians (interquartile ranges) and frequency (percentage). Differences in patient age and total GNPI score between different surgical groups were evaluated using the Kruskal–Wallis test with post hoc analysis of pairwise comparisons using the Bonferroni method. Differences in categorical variables (patient gender, tumour histology, tumour size, use of nasoseptal flap, prior endonasal surgery intra-operative CSF leak and post-operative meningitis/sinusitis) between groups were determined using Pearson’s chi-square test. To evaluate differences in total GNPI score at each time point between non-functioning pituitary adenomas (NFPAs) and functioning adenomas, the Mann–Whitney *U* test was used.

Changes in total GNPI score over time were analysed using a repeated measures mixed-effects model with imaging time point as a fixed-effects variable. Post hoc analysis of pairwise comparisons between different time points was performed using the Bonferroni method. Ordinal logistic regression was used to evaluate the effect of patient demographics (age, gender), surgical approach, tumour histology and tumour size on the total GNPI score pre-treatment and at post-operative days 1 and 3. Results are presented as odds ratios with 95% confidence intervals. In addition to comparison of total GNPI scores, the percentage of asymptomatic patients (score = 0) for each individual symptom question was reported and scores compared across each time point using Pearson’s chi-square test.

## Results

### Population characteristics and pathological data

There was no gender preponderance (female = 68; 50%). Median age of enrolled patients was 57.7 (IQR 44.5–70.0). Pituitary adenomas were the predominant pathology, present in 97 patients (71%) with remaining tumours as follows: 19 cystic lesions (14 Rathke’s cleft cyst and 5 craniopharyngiomas), 6 meningiomas and 14 miscellaneous (pituitary apoplexy, cholesterol granuloma, chordoma, lymphocytic hypophysitis, lymphoma, myeloma and Wegener’s granulomatosis). The majority of patients (*N* = 71; 52%) were diagnosed with NFPAs. The majority of lesions were macroadenomas (*N* = 83; 61%) with functioning tumours accounting for 19% (*N* = 26).

There were three subgroups based on surgical approaches used: 25 (18%) mTSS, 74 (54%) eTSS-uni and 37 (27%) eTSS-bi. Within the eTSS-uni approach group, three patients had undergone prior endonasal surgery for a NFPA. There were no revision surgeries within either the mTSS or eTSS-bi approach groups.

Intra-operative CSF leak was noted in 36% with no significant difference across either surgical groups (*p* > 0.05, chi-square test, Table 1). Repair of intra-operative CSF leak followed a graded repair utilizing non-vascularized autologous grafting and synthetic buttressing for low-grade leaks, and vascularized nasoseptal flaps for high-grade leaks [27]. No patients suffered from a post-operative CSF leak.

**Table 1:** Population characteristics and pathological data stratified by surgical approach

Factor		Total (%)	Microscopic TSS	Uninostril eTSS	Binostril eTSS	*p* value
***N***		136	25	74	37	
Median age, years (IQR)		57.7 (44.5–70.0)	54.4 (47.9–66.3)	59.4 (43.1–68.5)	57.0 (44.6–73.7)	0.99
Gender	Male	68 (50)	11 (44)	37 (50)	20 (54)	0.74
	Female	68 (50)	14 (66)	37 (50)	17 (46)	
Histology	NFPA	71 (52)	9 (36)	46 (62)	16 (43)	**0.02**
	Functioning (ACTH/GH/PRL)	26 (19)	5 (20)	14 (19)	7 (19)	
	Meningioma	6 (4)	0 (0)	1 (1)	5 (14)	
	RCC	14 (10)	4 (16)	8 (11)	2 (5)	
	Craniopharyngioma	5 (4)	2 (8)	1 (1)	2 (5)	
	Other*	14 (10)	5 (20)	4 (5)	5 (14)	
Tumour size	Macroadenoma	83 (61)	11 (44)	52 (70)	20 (54)	0.08
	Microadenoma	14 (10)	3 (12)	8 (11)	3 (8)	
	Non-adenoma	39 (29)	11 (44)	14 (19)	14 (38)	
Use of septal flap	Yes	23 (17)	4 (16)	4 (5)	15 (41)	** < 0.001**
Prior endonasal surgery	Yes	3 (2)	0 (0)	3 (4)	0 (0)	0.28
Intra-op CSF leak	Present	49 (36)	5 (20)	29 (39)	15 (41)	0.18

Absolute number and percentage shown (in brackets). *p* value calculated using Kruskal–Wallis test with post hoc analysis of pairwise comparisons using the Bonferroni method. Differences in patient gender, tumour histology, tumour size, use of nasoseptal flap and intra-operative CSF leak determined using Pearson’s chi-square test

*ACTH*, corticotropinoma; *eTSS*, endoscopic transsphenoidal surgery; *GH*, somatotropinoma; *IQR*, interquartile range; *NFPA*, non-functioning pituitary adenoma; *PRL*, prolactinoma; *RCC*, Rathke’s cleft cyst; *eTSS*, endoscopic transsphenoidal surgery

^*^Other = apoplexy; cholesterol granuloma; chordoma; lymphocytic hypophysitis; lymphoma; myeloma; Wegener’s granulomatosis

A nasoseptal flap repair was utilized significantly more in the eTSS-bi subgroup (*p* < 0.001, chi-square test). All 4 patients (100%) undergoing nasoseptal flap repair in the eTSS-uni group had documented intra-operative CSF leak compared to 2 patients (50%) in the mTSS group and 12 patients (80%) in the eTSS-bi group. There was a significant difference in the presenting pathology of patients undergoing nasoseptal repair (*p* < 0.001, chi-square test) with 5/6 patients undergoing meningioma resection requiring nasoseptal flap repair compared to only 7/71 patients with NFPA and 1/26 patients undergoing surgery for functioning tumours, respectively. After undergoing the eTSS-uni approach, two patients (1%) developed post-operative meningitis and three patients (2%) developed sinusitis. There were no cases of meningitis/sinusitis following either the mTSS or eTSS-bi approach but these differences between approaches were not statistically significant (*p* > 0.05, chi-square test).

### Changes in GNPI scores over time

**Fig. 1:**
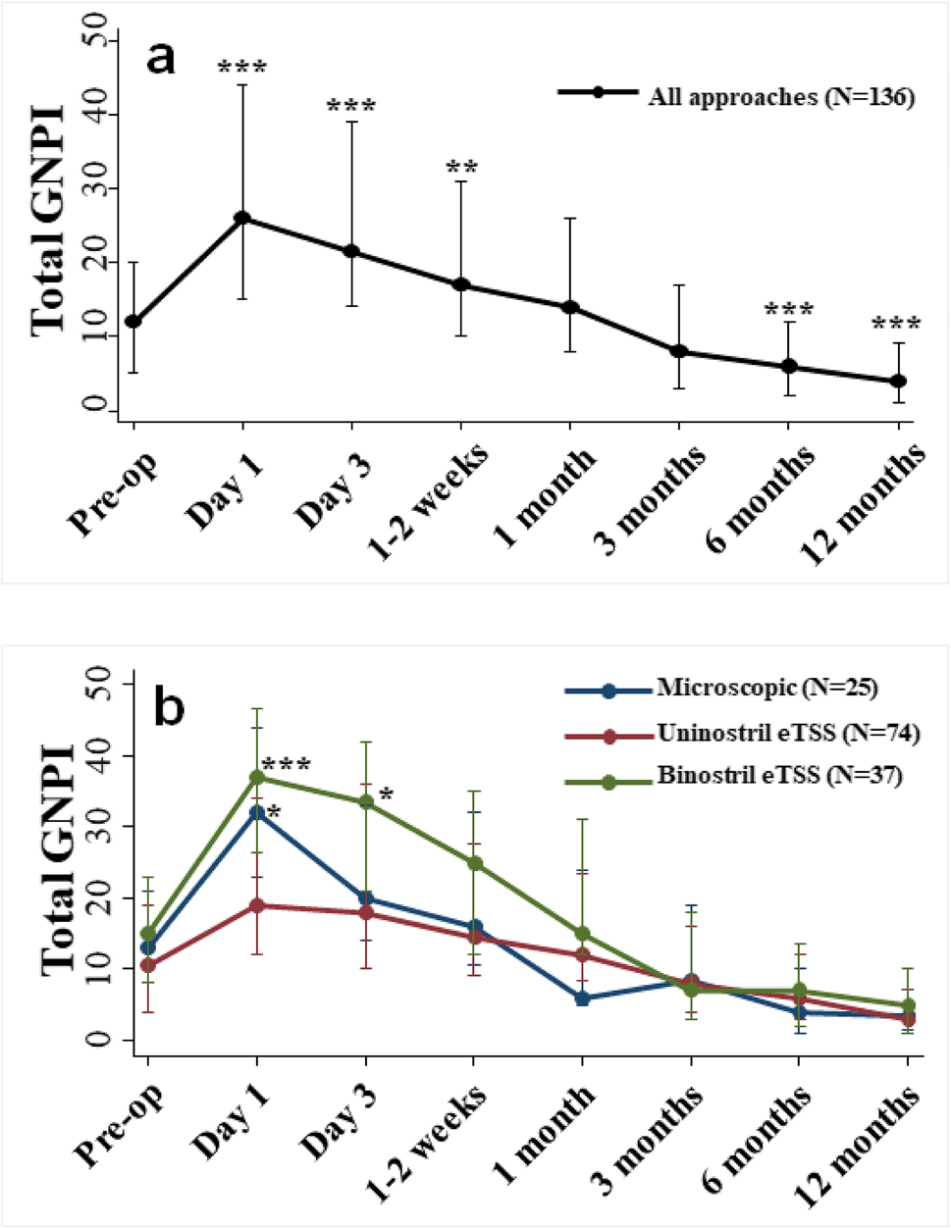
Total GNPI score changes over time stratified by surgical approach. Median and interquartile range of total GNPI score at each time point shown. **a** Change in total GNPI score over time for all 136 patients across all surgical approaches. Total GNPI scores were significantly higher than pre-treatment scores at post-operative day 1, day 3 and 1–2 weeks post-operatively and were significantly lower than pre-treatment scores at 6 months and 12 months post-treatment (mixed-effects model). **p ≤ 0.01; ***p ≤ 0.001. **b** Change in total GNPI score stratified by surgical approach. *p* value for microscopic TSS/binostril eTSS approach is shown and represents difference in GNPI score compared to uninostril eTSS approach at each time point. *p* value calculated using Kruskal–Wallis test with post hoc analysis of pairwise comparisons using the Bonferroni method. *p ≤ 0.05; **p ≤ 0.01; ***p ≤ 0.001. eTSS, endoscopic transsphenoidal surgery

Changes in total GNPI score across all 136 patients are shown in Fig. 1a. Irrespective of surgical approach, total GNPI scores were significantly higher at post-operative day 1 (*p* < 0.001) and day 3 (*p* ≤ 0.03) compared to pre-treatment scores (mixed-effects model). Post-operative GNPI scores at 1 month post-operatively were no different from pre-treatment (*p* > 0.05, mixed-effects model). In both the eTSS-uni and eTSS-bi groups, total GNPI was significantly lower at 6 months (*p* ≤ 0.02) and 12 months (*p* ≤ 0.03) compared to pre-treatment. Whilst scores at 6 months and 12 months in the mTSS group were also lower than pre-treatment, this difference was not statistically significant (*p* > 0.05, mixed-effects model).

The eTSS-uni group demonstrated significantly lower GNPI scores at day 1 post-op compared to the mTSS group (*p* = 0.05) and eTSS-bi group (*p* < 0.001, Kruskal–Wallis test, Fig. 1b) but by weeks 1–2, this effect had disappeared. This difference in early (day 1) post-treatment GNPI scores between approaches was maintained even after exclusion of the six patients undergoing meningioma resection (supplementary Fig. S1). Exclusion of patients undergoing nasoseptal flap repair led to lower immediate (days 1 and 3) post-operative scores in all groups and lower scores at 6 months and 1 year in the eTSS-bi group (supplementary Fig. S2).

**Fig. 2:**
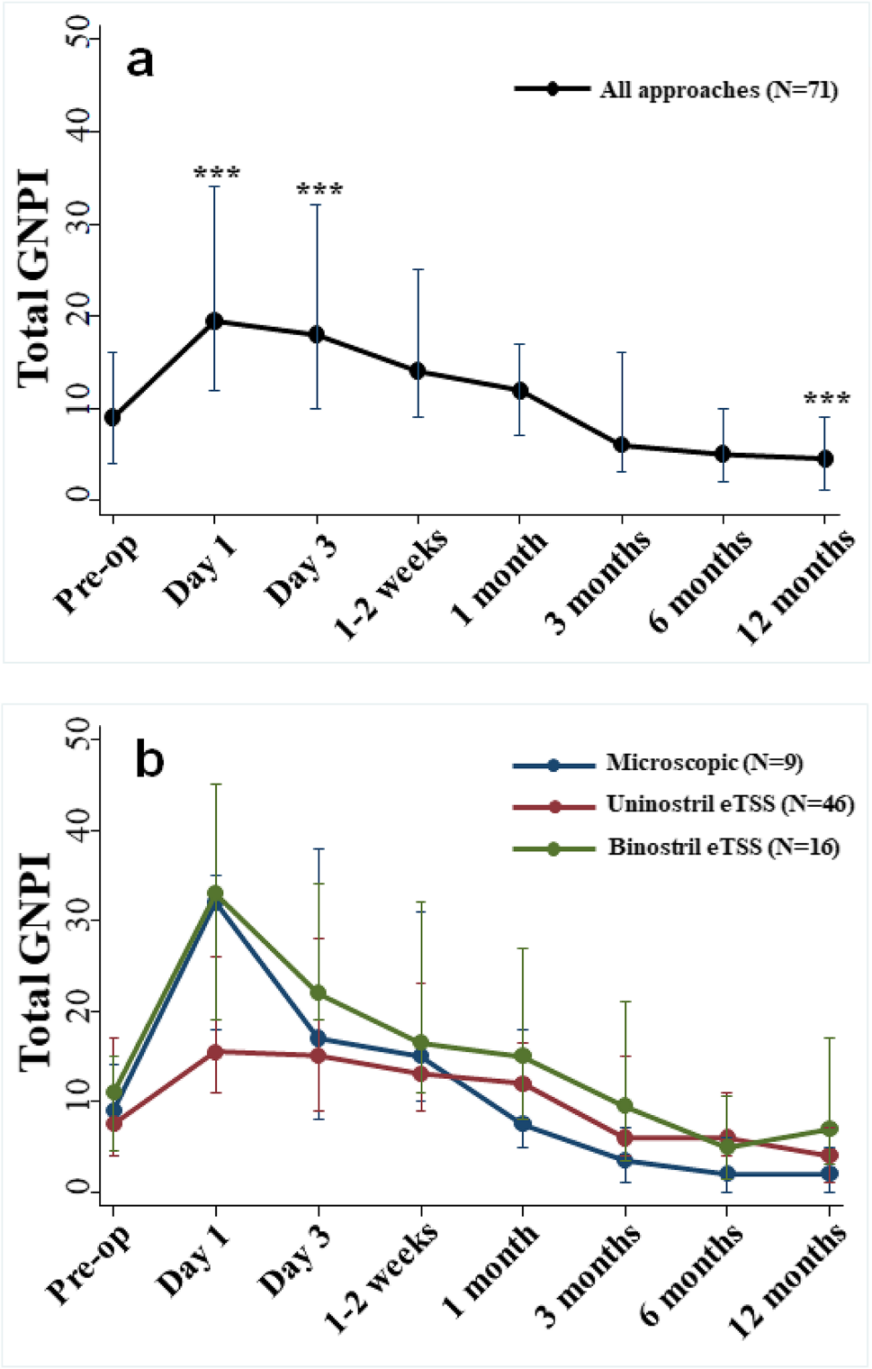
Total GNPI score changes over time stratified by surgical approach. Non-functioning pituitary adenoma (NFPA, *n* = 71) subgroup only. Median and interquartile range of total GNPI score at each time point shown. **a** Change in total GNPI score over time for all NFPA patients across all surgical approaches. Total GNPI scores within the subgroup of NFPA were significantly higher than pre-treatment scores at post-operative day 1 and day 3 and significantly lower than pre-treatment scores at 12 months post-treatment (mixed-effects model, ***p < 0.001). **b** Change in total GNPI score stratified by surgical approach. At post-operative day 1, the eTSS-uni group demonstrated lower GNPI scores compared to the mTSS group (median GNPI 16 vs 32) and eTSS-bi group (median GNPI 16 vs 33) but these results did not reach statistical significance (Kruskal–Wallis test with post hoc analysis of pairwise comparisons using the Bonferroni method). eTSS, endoscopic transsphenoidal surgery

Within the NFPAs (*N* = 71) subgroup, total GNPI scores were significantly higher than pre-treatment scores at post-operative day 1 (*p* < 0.001) and day 3 (*p* < 0.001) and significantly lower than pre-treatment scores at 12 months post-treatment (*p* < 0.001, mixed-effects model, Fig. 2a). Analysis of the NFPA subgroup demonstrated that at post-operative day 1, the eTSS-uni group demonstrated lower GNPI scores compared to the mTSS group (median GNPI 16 vs 32) and eTSS-bi group (median GNPI 16 vs 33) but these results did not reach statistical significance (*p* > 0.05, Kruskal–Wallis test, Fig. 2b).

As shown in supplementary table S1, the majority of GNPI items showed a significant trend with an initial drop in the percentage of asymptomatic patients (score = 0) up to 1 month post-operatively and a recovery in the percentage of asymptomatic patients close to or above pre-treatment levels by 6–12 months post-operatively. Specific items (e.g., “I get headaches,” “my sinuses are painful,” “my mouth is dry,” “my work is affected,” “I feel tired” and “I feel moody, depressed or irritable”) showed an overall improvement in the percentage of asymptomatic patients at 12 months post-operatively compared to pre-treatment.

### Comparison in GNPI scores between non-functioning and functioning tumours

**Fig. 3:**
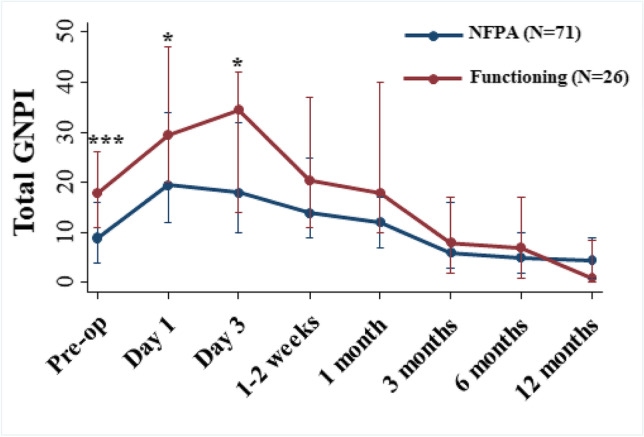
Total GNPI score changes over time stratified by adenoma functional status. Median and interquartile range of total GNPI score at each time point shown. *p* value represents difference in total GNPI score between non-functioning/functioning tumours at each time point. *p* value calculated using Mann–Whitney *U* test. *p ≤ 0.05; **p ≤ 0.01; ***p ≤ 0.001. ACTH, corticotropinoma; GH, somatotropinoma; NFPA, non-functioning pituitary adenoma; PRL, prolactinoma

Patients with functioning adenomas had higher GNPI scores at baseline (pre-treatment) compared to NFPA (*p* < 0.001, Mann–Whitney *U* test), but by week 1, this difference has disappeared (Fig. 3). Presence of a functioning tumour was a significant predictor of higher pre-treatment GNPI (OR 3.93, *p* = 0.007) but age, gender and tumour size were not significant predictors.

Ordinal logistic regression (Table 2) demonstrated that significant independent predictors of higher GNPI at day 1 post-op were younger age (OR 0.96, *p* = 0.001), higher pre-treatment GNPI (OR = 1.08, *p* < 0.001), microscopic rather than eTSS-uni (OR 0.30, *p* = 0.008) and the use of a nasoseptal flap (OR 3.48, *p* = 0.02). There was no predictive value of a functioning tumour resulting in higher GNPI at day 1 once the higher pre-treatment GNPI is accounted for (OR 0.48, *p* = 0.14).

**Table 2:** Ordinal logistic regression to evaluate the effect of each parameter on post-operative day 1 and day 3 GNPI score (*N* = 136)

Variable		Day 1 GNPI			Day 3 GNPI		
		OR	95% OR	***p***value	OR	95% OR	***p*** value
Age, years		**0.96**	**0.94, 0.99**	**0.001**	**0.98**	**0.96, 0.99**	**0.04**
Gender	Female	1.21	0.62, 2.36	0.57	**2.26**	**1.13, 4.53**	**0.02**
Pre-treatment GNPI		**1.08**	**1.05, 1.11**	** < 0.001**	**1.08**	**1.05, 1.10**	** < 0.001**
Approach base: Microscopic	Uninostril eTSS	**0.30**	**0.12, 0.72**	**0.008**	1.123	0.51, 2.97	0.64
	Binostril eTSS	1.09	0.42, 2.79	0.86	2.01	0.79, 5.10	0.14
Histology base: NFPA	Functioning (ACTH/GH/PRL)	0.48	0.18, 1.28	0.14	0.84	0.30, 2.31	0.74
	Meningioma	1.34	0.13, 13.4	0.80	1.51	0.19, 12.5	0.70
	RCC	0.31	0.06, 1.59	0.16	0.78	0.16, 3.66	0.75
	Craniopharyngioma	0.32	0.04, 2.72	0.29	0.66	0.09, 5.15	0.70
	Other*	**0.14**	**0.02, 0.75**	**0.02**	0.40	0.08, 2.10	0.28
Tumour size base: microadenoma	Macroadenoma	0.32	0.08, 1.20	0.09	0.74	0.21, 2.64	0.65
Use of septal flap	Yes	**3.48**	**1.18, 10.3**	**0.02**	**6.09**	**1.90, 19.4**	**0.002**
Intra-op CSF leak	Present	0.70	0.33, 1.47	0.35	0.70	0.33, 1.45	0.33

*ACTH*, corticotropinoma; *eTSS*, endoscopic transsphenoidal surgery; *GH*, somatotropinoma; *IQR*, interquartile range; *NFPA*, non-functioning pituitary adenoma; *PRL*, prolactinoma; *RCC*, Rathke’s cleft cyst; *eTSS*, endoscopic transsphenoidal surgery

^*^Other = apoplexy; cholesterol granuloma; chordoma; lymphocytic hypophysitis; lymphoma; myeloma; Wegener’s granulomatosis

## Discussion

There are few papers to directly compare nasal symptoms between established trans-nasal surgical approaches for pituitary region lesions. Previous studies focusing on singular approaches have confirmed an early increase of symptoms followed by resolution over the ensuing months. These studies utilized the GNPI or the 22-item sinonasal test (SNOT-22) investigating single-surgeon eTSS or mTSS, respectively [2, 25, 27]. The conclusions of these papers were that rhinological recovery is typically rapid and relatively complete by 3 to 4 months post-surgery [2, 4, 25, 27]. The implication of such studies was that the endoscopic approach affords a greater improvement in rhinological recovery, and this was further investigated in our study.

Contrary to previous reports, our study showed no differences in nasal symptoms beyond 1–2 weeks, when comparing mTSS, and eTSS-uni or eTSS-bi. Eseonu et al. have reported an economic benefit in eTSS over mTSS with a trend towards decreased nasal complications [5]. Most reported complications are however anatomical issues including septal perforations, nasal adhesions and epistaxis [7, 9, 17, 18, 24]. Using patient-reported questionnaires such as the GNPI and SNOT-22, the functional impact of such anatomical issues seems to be less significant. It has also been shown that the experience of the operating surgeon results in significant improvement in endocrinological outcomes following transsphenoidal surgery [10, 12, 26]. In this study, all surgeons involved were specialist trained pituitary surgeons having a combined surgical experience of over three thousand patients. A low rhinological complication rate from the included cohort of patients is therefore expected and a limitation of this study is that the findings may not be broadly applicable to lower volume treatment centres and practitioners.

Little et al. carried out a multi-modality review of mTSS compared to eTSS [19]. No specific patient-reported outcomes were assessed in their study; however, significant differences were found in favour of mTSS with regard to duration of surgery [19]. In our experience, the endoscopic technique requires a more careful dissection of the nasal cavity to allow the passage of instruments, whilst minimizing contamination of the endoscope lens with blood and debris. This may contribute to the increased duration of surgery undertaken with an endoscope. Our observation that the early post-operative nasal symptom scores following unilateral eTSS were marginally lower compared to mTSS would imply reduced nasal trauma secondary to static retraction by the nasal speculum in mTSS [21].

Our study also revealed a sustained improvement in post-operative nasal symptoms following eTSS-uni and eTSS-bi from 6 months post-surgery compared to pre-operative nasal scores. A similar trend was seen in the mTSS group, and whilst not statistically significant, this may reflect the lower number of patients in this group. These findings agree with a recent study utilizing patient-reported outcome measures to assess nasal symptoms in patients undergoing endoscopic and microscopic approaches [21]. Pledger et al. reported significant improvement across vitality, mental and physical health, and social functioning by 1 year after surgery in both mTSS and eTSS [21]. Improvements in nasal cavity anatomy, by addressing nasal polyps and septal deviations during the nasal phase of TSS, may in part contribute to the post-operative improvement in patient-related outcome [15]. Successful treatment of the patients’ underlying condition, notably surgical cure of functioning adenomas such as in acromegaly and Cushing’s disease, may also in part explain this trend [2].

In developed countries, endoscopic transsphenoidal surgery is increasingly becoming the standard approach for resection of pituitary lesions and this is evidenced by the larger number of patients undergoing eTSS compared to mTSS in our cohort [6, 20]. Implementation of endoscopic endonasal surgery in low- and middle-income countries, however, remains challenging and a comparison of post-operative sinonasal outcomes between microscopic and endoscopic approaches is therefore still relevant. Our study demonstrates that whilst early post-operative nasal symptom scores following unilateral eTSS are lower, long-term nasal outcomes across endoscopic and microscopic approaches are comparable. Whilst there are clear surgical benefits afforded by the endoscopic technique (Refs. 3–5), this equivalence in long-term patient quality of life measures between approaches should be recognized and appraised before implementing potentially costly endoscopic techniques in resource poor healthcare settings.

One interesting finding of our study was that patients with functioning adenomas had worse patient-reported symptoms at baseline (pre-treatment) compared to those with non-functioning tumours. This may reflect the fact that some functioning tumours (e.g., somatotropinoma) present with upper-airway and nasal symptoms [1, 6, 20, 22]. In acromegaly for example, growth hormone excess can lead to hypertrophy of nasal passages and pharyngeal tissues, leading to increased nasal symptoms and sleep apnea pre-operatively [6, 22]. Excess hormone production in functioning tumours such as in Cushing’ disease has similarly been associated with reduced general quality of life indices in earlier studies [5, 28]. The post-operative improvement seen in GNPI scores in the functioning group is therefore likely to reflect reduction in excess hormone production from these tumours as well as changes in nasal anatomy following surgery [1, 6, 20, 22].

In the present study, the use of the nasoseptal flap was noted to increase the post-operative nasal symptoms in the short-term. The efficacy of the nasoseptal flap for CSF leak repair is well established [14, 23] and although 36% of subjects had an intra-operative leak noted in our study, no patients suffered from a post-operative CSF leak. The absence of post-op CSF leaks in patients with low-grade intra-operative CSF leaks managed without flaps suggests it may be safe to omit the nasoseptal flap where there is no significant dural resection [27].

## Conclusion

Nasal symptoms following transsphenoidal surgery are mild and self-limiting regardless of surgical approach. Contrary to previous reports, the microscopic transsphenoidal approach does not result in worse long-term nasal outcomes compared to the endoscopic approach. Transsphenoidal surgery is well tolerated regardless of approach with potential for long-term improvement in nasal function beyond 6 months post-surgery.

## Supplementary Information

Below is the link to the electronic supplementary material.

Supplementary file1 (DOCX 159 KB)

## Abbreviations

TSS: Transsphenoidal surgery
mTSS: Microscopic transsphenoidal surgery
eTSS: Endoscopic transsphenoidal surgery
eTSS-uni: Endoscopic uninostril transsphenoidal surgery
eTSS-bi: Endoscopic binostril transsphenoidal surgery

## References

[1] Akbari H, Malek M, Ghorbani M, Ramak Hashemi SM, Khamseh ME, Zare Mehrjardi A, Emami Z, Ebrahim Valojerdi A (2018). Clinical outcomes of endoscopic versus microscopic trans-sphenoidal surgery for large pituitary adenoma. Br J Neurosurg.

[2] Davies BM, Tirr E, Wang YY, Gnanalingham KK (2017). Transient exacerbation of nasal symptoms following endoscopic transsphenoidal surgery for pituitary tumors: a prospective study. J Neurol Surg B, Skull base.

[3] Douglas SA, Marshall AH, Walshaw D, Robson AK, Wilson JA (2001). The development of a General Nasal Patient Inventory. Clin Otolaryngol Allied Sci.

[4] Dusick JR, Esposito F, Mattozo CA, Chaloner C, McArthur DL, Kelly DF (2006). Endonasal transsphenoidal surgery: the patient’s perspective-survey results from 259 patients. Surg Neurol.

[5] Eseonu CI, ReFaey K, Garcia O, Salvatori R, Quinones-Hinojosa A (2018). Comparative cost analysis of endoscopic versus microscopic endonasal transsphenoidal surgery for pituitary adenomas. J Neurol Surg B, Skull base.

[6] Eseonu CI, ReFaey K, Rincon-Torroella J, Garcia O, Wand GS, Salvatori R, Quinones-Hinojosa A (2017). Endoscopic versus microscopic transsphenoidal approach for pituitary adenomas: comparison of outcomes during the transition of methods of a single surgeon. World neurosurgery.

[7] Fang J, Xie S, Li N, Jiang Z (2018). Postoperative complications of endoscopic versus microscopic transsphenoidal pituitary surgery: a meta-analysis. J Coll Phys Surgeons-Pakistan.

[8] Fatemi N, Dusick JR, de Paiva Neto MA, Kelly DF (2008). The endonasal microscopic approach for pituitary adenomas and other parasellar tumors: a 10-year experience. Neurosurgery.

[9] Gao Y, Zhong C, Wang Y, Xu S, Guo Y, Dai C, Zheng Y, Wang Y, Luo Q, Jiang J (2014). Endoscopic versus microscopic transsphenoidal pituitary adenoma surgery: a meta-analysis. World J Surg Oncol.

[10] Gittoes NJ, Sheppard MC, Johnson AP, Stewart PM (1999). Outcome of surgery for acromegaly—the experience of a dedicated pituitary surgeon. QJM: Mon J Ass Phys.

[11] Hadad G, Bassagasteguy L, Carrau RL, Mataza JC, Kassam A, Snyderman CH, Mintz A (2006). A novel reconstructive technique after endoscopic expanded endonasal approaches: vascular pedicle nasoseptal flap. Laryngoscope.

[12] Honegger J, Grimm F (2018). The experience with transsphenoidal surgery and its importance to outcomes. Pituitary.

[13] Jane JA, Han J, Prevedello DM, Jagannathan J, Dumont AS, Laws ER (2005). Perspectives on endoscopic transsphenoidal surgery. Neurosurg Focus.

[14] Kassam AB, Prevedello DM, Carrau RL, Snyderman CH, Thomas A, Gardner P, Zanation A, Duz B, Stefko ST, Byers K, Horowitz MB (2011). Endoscopic endonasal skull base surgery: analysis of complications in the authors’ initial 800 patients. J Neurosurg.

[15] Kim DH, Hong YK, Jeun SS, Park YJ, Kim SW, Cho JH, Kim BY, Han S, Lee YJ, Hwang JH, Kim SW (2016). Intranasal volume changes caused by the endoscopic endonasal transsphenoidal approach and their effects on nasal functions. PloS One.

[16] Kiraz M, Gunaldi O, Tanriverdi O, Erdim I, Postalci LS, Tugcu B, Yazici MZ (2018). Comparison of sinonasal complications of microscopic and endoscopic approaches for transsphenoidal hypophyseal surgery: prospective study. Turk Neurosurg.

[17] Li A, Liu W, Cao P, Zheng Y, Bu Z, Zhou T (2017). Endoscopic versus microscopic transsphenoidal surgery in the treatment of pituitary adenoma: a systematic review and meta-analysis. World Neurosurg.

[18] Li J, Ding W, Huang Z, Xie B, Li ZY (2019). Comparison of short-term outcomes between endoscopic and microscopic trans-sphenoidal surgery for the treatment of pituitary adenoma. J Craniofac Surg.

[19] Little AS, Kelly DF, Milligan J, Griffiths C, Prevedello DM, Carrau RL, Rosseau G, Barkhoudarian G, Jahnke H, Chaloner C, Jelinek KL, Chapple K, White WL (2015). Comparison of sinonasal quality of life and health status in patients undergoing microscopic and endoscopic transsphenoidal surgery for pituitary lesions: a prospective cohort study. J Neurosurg.

[20] Little AS, Kelly DF, White WL, Gardner PA, Fernandez-Miranda JC, Chicoine MR, Barkhoudarian G, Chandler JP, Prevedello DM, Liebelt BD, Sfondouris J, Mayberg MR (2019) Results of a prospective multicenter controlled study comparing surgical outcomes of microscopic versus fully endoscopic transsphenoidal surgery for nonfunctioning pituitary adenomas: the Transsphenoidal Extent of Resection (TRANSSPHER) study. J Neurosurg 1–11 10.3171/2018.11.JNS181238 30901746

[21] Pledger CL, Elzoghby MA, Oldfield EH, Payne SC, Jane JA (2016). Prospective comparison of sinonasal outcomes after microscopic sublabial or endoscopic endonasal transsphenoidal surgery for nonfunctioning pituitary adenomas. J Neurosurg.

[22] Prajapati HP, Jain SK, Sinha VD (2018). Endoscopic versus microscopic pituitary adenoma surgery: an institutional experience. Asian J Neurosurg.

[23] Simal-Julián JA, Miranda-Lloret P, de San P, Román Mena L, Sanromán-Álvarez P, García-Piñero A, Sanchis-Martín R, Botella-Asunción C, Kassam A (2020). Impact of multilayer vascularized reconstruction after skull base endoscopic endonasal approaches. J Neurol Surg B, Skull base.

[24] Strychowsky J, Nayan S, Reddy K, Farrokhyar F, Sommer D (2011). Purely endoscopic transsphenoidal surgery versus traditional microsurgery for resection of pituitary adenomas: systematic review. J Otolaryngol Head Neck Surg = Le Journal d’oto-rhino-laryngologie et de chirurgie cervico-faciale.

[25] Wang S, Chen Y, Li J, Wei L, Wang R (2015). Olfactory function and quality of life following microscopic endonasal transsphenoidal pituitary surgery. Medicine.

[26] Wang YY, Higham C, Kearney T, Davis JR, Trainer P, Gnanalingham KK (2012). Acromegaly surgery in Manchester revisited—the impact of reducing surgeon numbers and the 2010 consensus guidelines for disease remission. Clin Endocrinol.

[27] Wang YY, Srirathan V, Tirr E, Kearney T, Gnanalingham KK (2011). Nasal symptoms following endoscopic transsphenoidal pituitary surgery: assessment using the General Nasal Patient Inventory. Neurosurg Focus.

[28] Yu SY, Du Q, Yao SY, Zhang KN, Wang J, Zhu Z, Jiang XB (2018). Outcomes of endoscopic and microscopic transsphenoidal surgery on non-functioning pituitary adenomas: a systematic review and meta-analysis. J Cell Mol Med.

[29] Zador Z, Gnanalingham K (2013). Endoscopic transnasal approach to the pituitary—operative technique and nuances. Br J Neurosurg.

