# Supplementary material for: Equivalent outcomes in nasal symptoms following microscopic or endoscopic transsphenoidal surgery: results from multi-centre, prospective study
Source: Acta Neurochir (Wien). 2022 Feb 8;164(6):1589–97. doi: 10.1007/s00701-022-05138-5 (PMC9160117; doi:10.1007/s00701-022-05138-5)
Supplement: Supplementary file 1 — Supplementary file1 (DOCX 159 KB) [file 701_2022_5138_MOESM1_ESM.docx]

**EQUIVALENT OUTCOMES IN NASAL SYMPTOMS FOLLOWING MICROSCOPIC OR ENDOSCOPIC TRANSSPHENOIDAL SURGERY: RESULTS FROM MULTI-CENTRE, PROSPECTIVE STUDY**

Charlie Osborne^1^ MD*, Daniel Lewis^2^ PhD*, Ben Dixon^3^ PhD, Carmela Caputo^4^ MBBS, Alison Magee^5^ RN, Kanna Gnanalingham^2^ PhD, Yi Yuen Wang^1,2,5^ MD

*^1^Department of Neurosurgery, St Vincent’s Hospital, Melbourne, Victoria, Australia*

*^2^Department of Neurosurgery, Manchester Centre for Clinical Neurosciences, Salford Royal NHS foundation trust, Manchester Academic Health Science Centre, Manchester, United Kingdom*

*^3^Department of Ear, Nose & Throat, Head and Neck Surgery, St Vincent’s Hospital, Melbourne, Victoria, Australia*

*^4^Department of Endocrinology, St Vincent’s Hospital, Melbourne, Victoria, Australia*

*^5^Keyhole Neurosurgery, Melbourne, Victoria, Australia*

Corresponding author: Yi Yuen Wang, Department of Neurosurgery, St Vincent’s Hospital

### Email: y.wang@keyholeneurosurgery.com.au

Journal name: Acta Neurochirurgica – The European Journal of Neurosurgery

# **Supplementary figures**


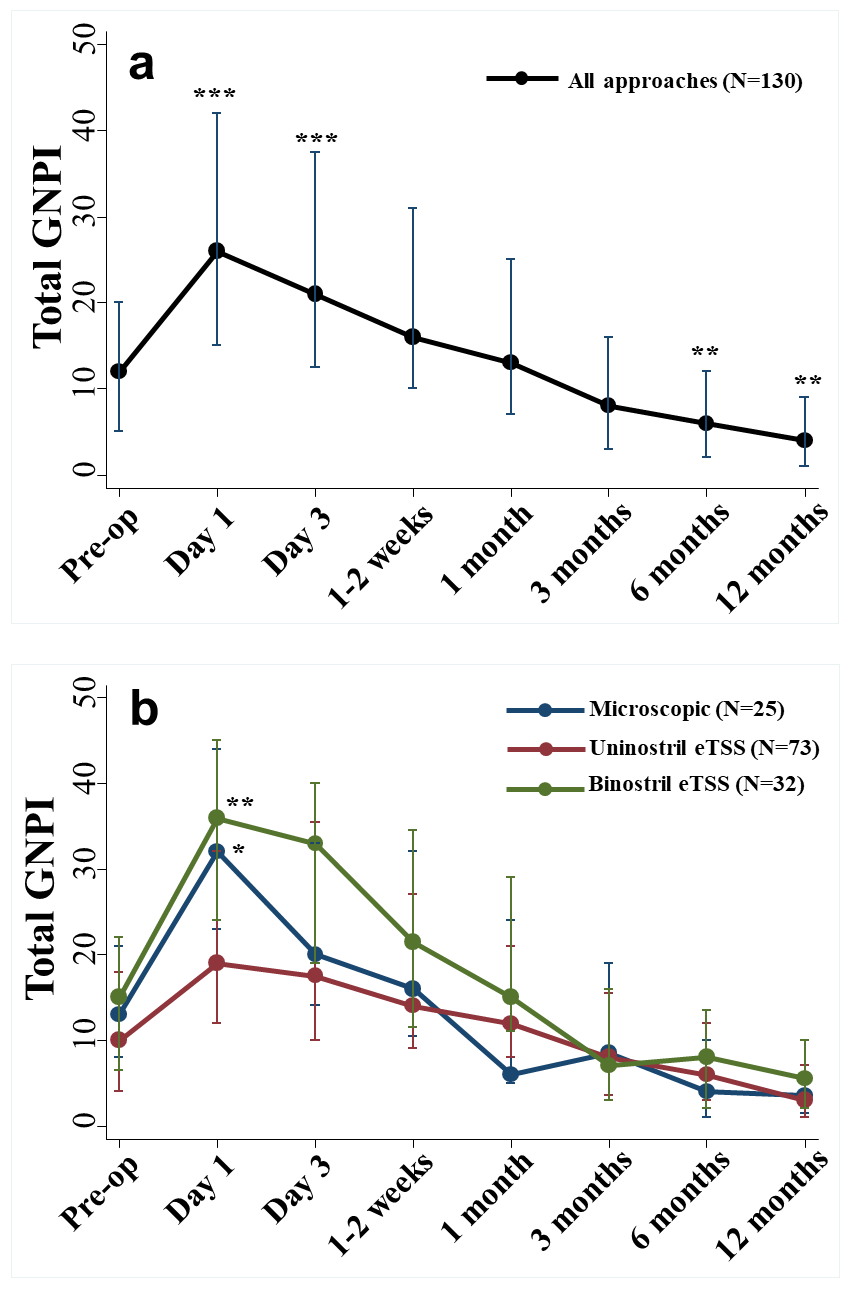


**Figure S1: Total GNPI score changes over time stratified by surgical approach (six patients with meningioma excluded)**

Median and interquartile range of total GNPI score at each timepoint shown.

A: Change in total GNPI score over time for all 130 patients across all surgical approaches. Total GNPI scores were significantly higher than pre-treatment scores at post-operative day 1 and day 3 post-operatively and were significantly lower than pre-treatment scores at 6 months and 12 months post-treatment (mixed-effects model). ** P≤ 0.01; ***P≤ 0.001.

B: Change in total GNPI score stratified by surgical approach. P value for microscopic TSS / binostril eTSS approach is shown and represents difference in GNPI score compared to uninostril eTSS approach at each timepoint. P value calculated using Kruskal-Wallis test with post hoc analysis of pairwise comparisons using the Bonferroni method. * P ≤ 0.05; ** P ≤ 0.01; *** P ≤ 0.001

eTSS = endoscopic transsphendoidal surgery


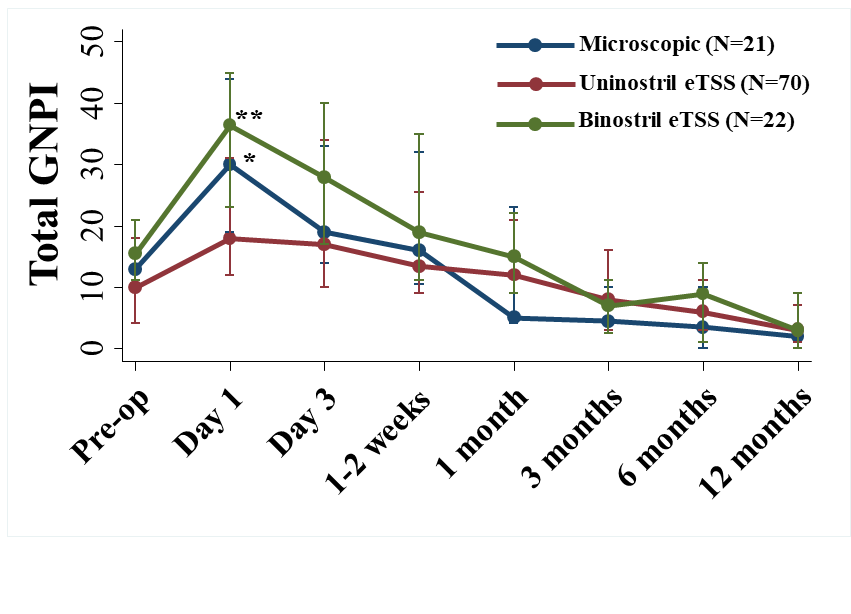


**Figure S2: Total GNPI score changes over time stratified by surgical approach (patients undergoing nasoseptal flap excluded)**

Median and interquartile range of total GNPI score at each timepoint shown. P value for microscopic/ binostril eTSS approach is shown and represents difference in GNPI score compared to uninostril eTSS approach. P value calculated using Kruskal-Wallis test with post hoc analysis of pairwise comparisons using the Bonferroni method. ** P ≤ 0.05; ** P ≤ 0.01; *** P ≤ 0.001*

*eTSS=endoscopic transsphenoidal surgery*

# **Supplementary tables**

**Table S1: Evolution of individual GNPI symptoms over time**

Percentage of asymptomatic patients for each symptom at each time point shown. P value indicates whether there is a significant change in all scores for an individual symptom over time and was calculated using Pearson’s chi-square test. Significant p values highlighted in bold. Data shown for all patients (N=136).

*GNPI= General Nasal Patient Inventory*

| **Symptom** | **Percentage (%) of asymptomatic patients at each time point.** | | | | | | | | **P value** |
| --- | --- | --- | --- | --- | --- | --- | --- | --- | --- |
|  | **Pre-** | **Day 1** | **Day 3** | **1-2 weeks** | **1 month** | **3 months** | **6 months** | **12 months** |  |
| **I have sores inside my nose** | 89.7 | 64.4 | 61.2 | 66.2 | 61.6 | 82.0 | 90.0 | 89.5 | **<0.001** |
| **I get headaches** | 47.8 | 32.6 | 33.6 | 37.6 | 42.5 | 63.1 | 63.6 | 71.1 | **<0.001** |
| **I take too many painkillers** | 72.8 | 61.5 | 63.9 | 70.7 | 72.6 | 84.4 | 85.5 | 84.2 | **<0.001** |
| **There is an unpleasant smell in my nose** | 91.9 | 91.9 | 89.6 | 82.7 | 74.0 | 86.1 | 90.0 | 90.8 | **0.003** |
| **My nose bleeds** | 86.0 | 29.6 | 55.2 | 75.9 | 83.6 | 91.8 | 96.4 | 96.1 | **<0.001** |
| **My sinuses are painful** | 77.2 | 73.3 | 68.7 | 69.9 | 78.1 | 86.1 | 91.8 | 92.1 | **<0.001** |
| **My nose is blocked** | 71.3 | 20.7 | 19.4 | 33.1 | 45.2 | 73.0 | 86.4 | 84.2 | **<0.001** |
| **I feel dripping at the back of my nose** | 85.3 | 62.2 | 70.1 | 70.7 | 71.2 | 83.6 | 86.4 | 92.1 | **<0.001** |
| **I have an unpleasant taste in my mouth** | 81.6 | 71.9 | 67.9 | 69.9 | 74.0 | 80.3 | 86.4 | 88.2 | **0.03** |
| **I have feelings of nausea** | 73.5 | 60.7 | 68.7 | 77.4 | 79.5 | 86.9 | 90.0 | 90.8 | **<0.001** |
| **My mouth is dry** | 61.0 | 21.5 | 24.6 | 41.4 | 64.4 | 69.7 | 75.5 | 81.3 | **<0.001** |
| **My nose feels uncomfortable** | 83.1 | 34.1 | 34.3 | 50.4 | 52.1 | 79.5 | 84.5 | 89.5 | **<0.001** |
| **My taste is affected** | 83.8 | 55.6 | 52.2 | 52.6 | 45.2 | 68.9 | 72.7 | 78.9 | **<0.001** |
| **My work is affected** | 70.6 | 59.3 | 56.8 | 54.9 | 51.4 | 75.4 | 81.8 | 94.7 | **<0.001** |
| **My voice changes** | 78.7 | 51.9 | 58.2 | 64.7 | 76.7 | 84.4 | 90.0 | 90.8 | **<0.001** |
| **My jaws are sore** | 83.8 | 85.2 | 88.1 | 86.5 | 86.3 | 93.4 | 96.4 | 97.4 | **0.03** |
| **I feel tired** | 37.5 | 20.0 | 18.7 | 23.3 | 26.0 | 40.2 | 47.3 | 61.8 | **<0.001** |
| **I have sore ears** | 82.4 | 83.7 | 87.3 | 88.7 | 86.3 | 90.2 | 96.4 | 92.1 | 0.17 |
| **My nose makes unusual noises** | 91.1 | 68.9 | 69.4 | 75.9 | 84.9 | 89.3 | 91.8 | 96.1 | **<0.001** |
| **My sleep is disturbed** | 50.7 | 25.2 | 31.3 | 42.9 | 47.9 | 65.6 | 64.5 | 73.7 | **<0.001** |
| **My nose is painful to touch** | 94.9 | 63.7 | 67.9 | 74.4 | 78.1 | 91.0 | 92.7 | 93.4 | **<0.001** |
| **My sense of smell is affected** | 77.2 | 35.6 | 40.3 | 37.6 | 38.4 | 63.9 | 64.5 | 72.4 | **<0.001** |
| **I have a choking feeling** | 89.7 | 78.5 | 87.3 | 89.5 | 86.3 | 93.4 | 97.3 | 96.1 | **0.004** |
| **My nose looks out of shape** | 86.0 | 81.5 | 85.1 | 89.5 | 87.5 | 95.1 | 94.5 | 96.0 | **0.01** |
| **I have sneezing attacks** | 75.0 | 85.2 | 76.1 | 82.0 | 69.9 | 82.8 | 84.5 | 89.5 | 0.09 |
| **I have pains in my face** | 81.5 | 79.3 | 78.4 | 82.0 | 83.6 | 91.8 | 95.5 | 94.7 | **0.004** |
| **I have to breathe through my mouth** | 83.0 | 23.7 | 27.6 | 39.8 | 61.6 | 84.4 | 91.8 | 92.1 | **<0.001** |
| **I suffer from hayfever** | 73.5 | 80.7 | 84.3 | 86.5 | 80.8 | 86.9 | 84.5 | 86.8 | 0.71 |
| **I have difficulty breathing** | 89.7 | 60.7 | 67.2 | 77.4 | 74.0 | 90.2 | 95.5 | 97.4 | **<0.001** |
| **I have difficulty talking or eating** | 91.2 | 65.9 | 73.1 | 81.2 | 83.6 | 91.8 | 97.3 | 96.1 | **<0.001** |
| **I have sore watering eyes** | 67.6 | 66.7 | 70.9 | 75.2 | 78.1 | 81.1 | 81.8 | 92.1 | **0.02** |
| **I feel moody, depressed, or irritable** | 58.8 | 74.1 | 67.2 | 64.7 | 65.8 | 73.8 | 78.2 | 78.9 | **0.01** |
| **My nose feels itchy** | 83.8 | 83.7 | 81.3 | 78.9 | 84.9 | 86.1 | 92.7 | 90.8 | 0.21 |
| **I am constantly sniffing** | 81.6 | 62.2 | 60.4 | 62.4 | 67.1 | 83.6 | 88.2 | 86.7 | **<0.001** |
| **My hearing is affected** | 80.1 | 82.2 | 88.1 | 87.2 | 86.3 | 85.2 | 90.9 | 93.3 | 0.54 |
| **I speak through my nose** | 89.0 | 64.4 | 64.9 | 72.2 | 80.8 | 86.1 | 88.2 | 93.4 | **<0.001** |
| **I have a sore throat** | 87.5 | 56.3 | 73.1 | 82.7 | 89.0 | 92.6 | 97.3 | 94.7 | **<0.001** |
| **My gums bleed** | 82.4 | 91.9 | 94.0 | 94.7 | 86.3 | 95.9 | 97.3 | 98.7 | **0.001** |
| **I feel dizzy** | 65.4 | 56.3 | 59.7 | 63.2 | 67.1 | 81.1 | 80.9 | 77.6 | **<0.001** |
| **I suffer from a cough** | 79.4 | 67.4 | 72.9 | 82.7 | 87.7 | 86.9 | 90.0 | 89.5 | **<0.001** |
| **People ask me if I have a cold** | 82.4 | 76.3 | 69.9 | 75.2 | 71.2 | 84.4 | 85.5 | 90.8 | **0.01** |
| **My nose runs** | 78.7 | 37.8 | 39.6 | 60.9 | 61.6 | 86.1 | 88.2 | 89.5 | **<0.001** |
| **I snore** | 41.9 | 40.0 | 44.0 | 48.9 | 50.7 | 54.9 | 55.5 | 60.5 | **0.04** |
| **I have bad breath** | 74.3 | 71.9 | 79.1 | 74.4 | 80.8 | 86.9 | 90.0 | 93.4 | **0.03** |
| **I get toothache** | 78.7 | 84.4 | 86.5 | 86.5 | 86.3 | 89.3 | 95.5 | 94.7 | **0.03** |
